# Beyond bold versus shy: Zebrafish exploratory behavior falls into several behavioral clusters and is influenced by strain and sex

**DOI:** 10.1242/bio.059443

**Published:** 2022-08-30

**Authors:** Neha Rajput, Kush Parikh, Justin W. Kenney

**Affiliations:** Department of Biological Sciences, Wayne State University, Detroit, MI 48202, USA

**Keywords:** Zebrafish, Behavior, Personality, Individual differences, Novel tank test, Exploratory behavior

## Abstract

Individual differences in exploratory behavior have been found across a range of taxa and are thought to contribute to evolutionary fitness. Animals that explore more of a novel environment and visit areas of high predation risk are considered bold, whereas animals with the opposite behavioral pattern are shy. Here, we determined whether this bimodal characterization of bold versus shy adequately captures the breadth of behavioral variation in zebrafish or if there are more than these two subtypes. To identify behavioral categories, we applied unsupervised machine to three-dimensional swim traces from over 400 adult zebrafish across four strains (AB, TL, TU, and WIK) and both sexes. We found that behavior stratified into four distinct clusters: previously described bold and shy behavior and two new behavioral types we call wall-huggers and active explorers. Clusters were stable across time and influenced by strain and sex where we found that TLs were shy, female TU fish were bold, male TU fish were active explorers, and male ABs were wall-huggers. Our work suggests that zebrafish exploratory behavior has greater complexity than previously recognized and lays the groundwork for the use of zebrafish in understanding the biological basis of individual differences in behavior.

## Abstract (precis)

**Summary:** Prior work described individual differences in zebrafish exploratory behavior as bold or shy. Here, we find four categories better describe their behavior: bold, shy, active explorers, and wall-huggers.

## INTRODUCTION

Interest in the biology of behavioral differences can be traced back to at least Greek and Roman antiquity where the humoral theory was used to explain variation in human temperament (Singer, 1928). Today, we have a greater understanding of human personalities, defined as behavioral tendencies that are consistent across time and context, but their biological basis remains elusive. One avenue for progress is the modeling of human personality through the study of individual differences in animal behavior. Often dismissed as noise around an average, a growing body of work has found that variations in animal behavior are often consistent across time and context (Dall et al., 2012; Sih et al., 2004). Such differences have been described for behaviors important for evolutionary fitness, and in a wide range of taxa, suggesting that they are conserved and provide grist for adaptation to an ever-changing environment.

One of the most widely studied axes of behavioral variation in animals is the bold–shy axis. Bold animals tend to explore or investigate novel environments or objects more readily than shy animals, which tend to flee or retreat in response to novelty (Réale et al., 2007; Toms et al., 2010; Wilson et al., 1994). From a fitness perspective, boldness may be adaptive when food resources are scarce and predation risk is low, whereas shyness may be more effective when the opposite conditions prevail (Réale et al., 2007). Variation along this axis has been described in animals ranging from bears (Myers and Young, 2018), lizards (López et al., 2005), birds (Carere et al., 2005), and fish (Toms et al., 2010). Studies examining the bold–shy axis typically begin with the assumption that animals fall into one of these two categories. However, it is unknown whether this bimodal distribution of bold versus shy fully captures variation in exploratory behavior. Indeed, recent work suggests that that there is more complexity to animal behavioral types, which may have been overlooked due to use of small sample sizes or assessment of only one or two specific behaviors (Forkosh et al., 2019). Identifying the presence of different behavioral types is a prerequisite for fully understanding how biological factors may contribute to the presence of individual differences in behavior.

Zebrafish have proven to be an excellent model organism to understand behavior and its biological basis. With 70% of fish genes having an obvious human ortholog (Howe et al., 2013), and a central nervous system that has the same general organization and uses many of the same neurotransmitters as mammals (Kenney et al., 2021; Panula et al., 2010; Wulliman et al., 1996), findings using zebrafish are widely applicable. Our understanding of zebrafish behavior has expanded rapidly over the past decade (Gerlai, 2020; Kalueff et al., 2013; Kenney, 2020), including several studies that have examined the bold–shy axis. Boldness is often probed by exposing fish to a novel tank and examining locomotion or avoidance behaviors, like geotaxis (i.e. bottom dwelling) or thigmotaxis (i.e. proximity to tank walls) (Mustafa et al., 2019; Oswald et al., 2012; Thörnqvist et al., 2019; Toms et al., 2010). Animals that are more active or spend more time in parts of the tank that would increase risk of predation (i.e. the top and/or center of the tank) are considered bolder. In zebrafish, these individual behaviors have been found to be consistent over time (Baker et al., 2018; Tran and Gerlai, 2013) and are predictive of other behaviors like social dominance (Dahlbom et al., 2011), aggression (Martins and Bhat, 2014), and stress reactivity (Oswald et al., 2012), all hallmarks of personality. However, fully using zebrafish exploratory behavior to understand the biological basis of individual differences requires that we first determine if this bold versus shy distinction adequately captures the breadth of behavioral variability exhibited during exploration.

To determine the presence of multiple behavioral clusters during exploration of a novel tank, we captured three-dimensional swim traces from over four hundred fish. Because zebrafish behavior is known to be influenced by strain and sex (Volgin et al., 2019), we used animals from four inbred strains (AB, TU, WIK, and TL) and both sexes to ensure that we captured the full range of behavioral variability. Using an unsupervised-machine-learning approach, we found that exploratory behavior stratified into four distinct clusters. These clusters included traditional descriptions of bold and shy, as well as two additional behavioral types that we dub wall-huggers and active explorers. Consistent with these behavioral subtypes being akin to personality types, we found that individual cluster membership remained largely consistent across days and weeks, and that the proportion of fish in each cluster was influenced by strain and sex.

## RESULTS

### Three-dimensional behavioral tracking

**Fig. 1.**
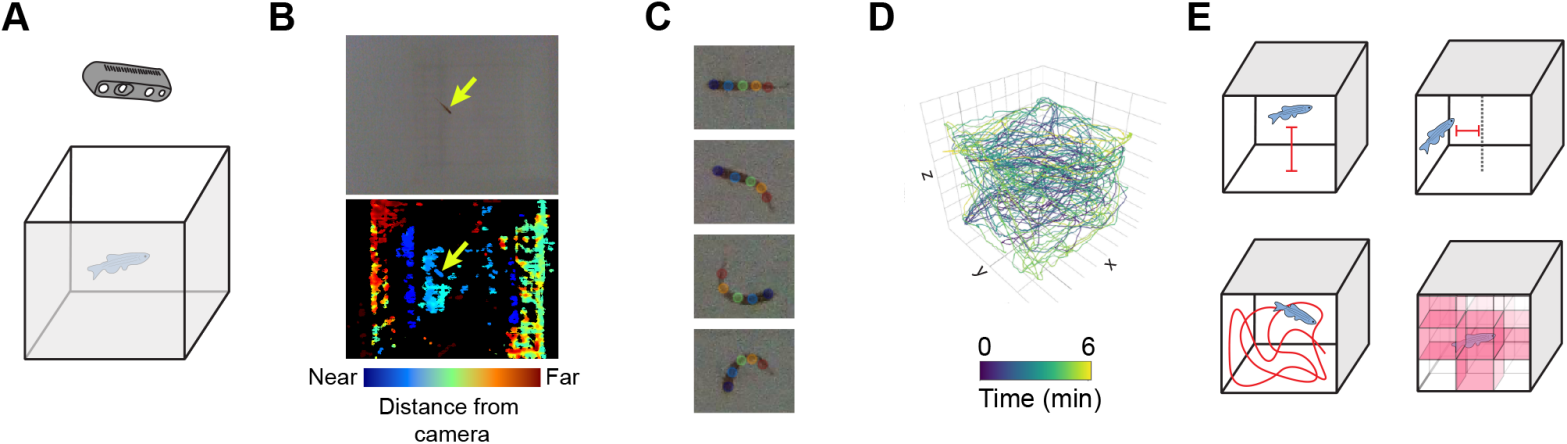
**Overview of three-dimensional behavioral tracking.** (A) Individual fish were placed into a novel tank while video was recorded from above using D435 Intel RealSense™ cameras. (B) Videos included both a color (top) and a depth (bottom) stream where fish can be seen (yellow arrows). (C) Animals were tracked in the color videos using DeepLabCut to identify five points along the length of the fish. (D) Tracking was overlaid with the depth stream to generate a three-dimensional trace for each animal. (E) Four exploratory parameters were extracted from each trace: bottom distance (top left), center distance (top right), distance travelled (bottom left), and percent of the tank explored (bottom right).

To capture three-dimensional zebrafish swim behavior during exploration of a tank, we used Intel RealSense™ cameras mounted above five-sided tanks with frosted walls (Fig. 1A). These cameras capture synchronized color and depth streams, resulting in three-dimensional videos (Fig. 1B). Fish posture at each frame was tracked in the color stream using DeepLabCut (Fig. 1C; Mathis et al., 2018). These points were overlayed onto the depth stream to create three-dimensional swim traces (Fig. 1D) from which we extracted positional information (distance from bottom and center), distance travelled, and percent of tank explored (Fig. 1E).

### Influence of sex and strain on exploratory behaviors

**Fig. 2.**
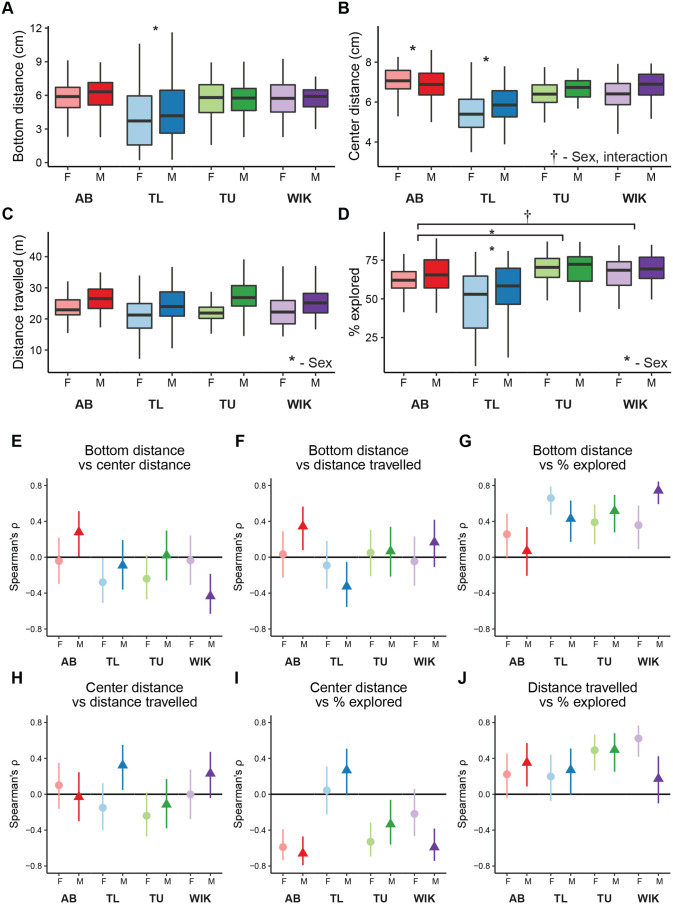
**Influence of sex and strain on individual exploratory behaviors.** The effect of sex and strain on (A) bottom distance, (B) center distance, (C) distance travelled, and (D) percent of the tank explored. Boxplots indicate median (center line), interquartile range (box ends), and hinge±1.5 times the interquartile range (whiskers). Spearman's rank correlation coefficient (ρ) with 95% confidence intervals across strain and sex for (E) bottom distance versus center distance, (F) bottom distance versus distance travelled, (G) bottom distance versus percent explored, (H) center distance versus distance travelled, (I) center distance versus percent explored, and (J) distance travelled versus percent explored. *P<0.05, †*P*<0.10 compared to all other groups or those indicated. AB, female: *n*=58, male: *n*=52; TL, female: *n*=54, male: *n*=50; TU, female: *n*=58, male: *n*=50; WIK, female: *n*=51, male: *n*=53.

We first determined whether sex or genetics influence individual zebrafish exploratory behaviors by assessing swim traces of fish from four strains (AB, TU, TL, and WIK), and both sexes, on 2 consecutive days. We extracted four exploratory behaviors from each swim trace: distance from bottom, distance from center, total distance travelled, and percentage of tank explored (Fig. 1E). We found that the distribution of several of the parameters deviated from normality (Fig. S1), so we performed non-parametric 4×2 (strain×sex) permutation ANOVAs. For distance from bottom (Fig. 2A), we found a main effect of strain (*P*=0.0001), but no effect of sex (*P*=0.41) or an interaction (*P*=0.45). False discovery rate (FDR)-corrected permutation *t*-tests found that TL fish swam closer to the bottom of the tank than all other strains (*P*’s≤0.0008). For distance from center (Fig. 2B) there was a trend towards a main effect of sex (*P*=0.053) and a trend towards an interaction (*P*=0.060) where female fish of every strain, except AB, spent more time closer to the center of the tank. We also found a main effect of strain (*P*=0.0001) with FDR corrected permutation *t*-tests indicating that TL fish swam closer to the center of the tank than all other strains (*P*’s=0.0004), and AB fish spent more time on the periphery (ABs compared to TU: *P*=0.0006, WIK: *P*=0.005). For distance travelled (Fig. 2C), we found a main effect of sex (*P*=0.0001) where male fish swam further than female fish. There was a trend towards an effect of strain (*P*=0.081), and no interaction (*P*=0.63). Finally, for percent of the tank explored (Fig. 2D), we also found a main effect of sex (*P*=0.018) in which female fish explored less of the tank than their male counterparts. There was also a main effect of strain (*P*=0.0001), but no interaction (*P*=0.40). Post-hoc tests revealed that TL fish explored the tank less than all other strains (*P*'s=0.0004), and that AB fish explored less than TU (*P*=0.046) with a trend towards a difference compared to WIK (*P*=0.088) fish. Taken together, we find that there are several sex differences (center distance, distance travelled, and percent explored) and that TL fish differ the most from other strains across all measures with no clear strain by sex interactions.

### Influence of sex and strain on within-session habituation to the tank

Some exploratory behaviors have been found to habituate over a single 6-min exposure to a novel tank (Wong et al., 2010), so we examined whether any of the parameters we measured changed over time and were influenced by sex in the various strains (Fig. S2). For each measure, we used non-parametric 2×6 (sex×time interval) mixed permutation ANOVAs and adjusted for multiple tests using an FDR correction. For distance from bottom (Fig. S2A), we found no main effects of sex (AB: *P*=0.43, TL: *P*=0.43, TU: *P*=0.66, WIK: *P*=0.85), but found effects of interval in all strains (AB: *P*=0.0006, TL: *P*=0.006, TU: *P*=0.0006, WIK: *P*=0.0012), where all fish, except WIKs, increased their distance from the bottom over time. There were no interactions except for WIKs (AB: *P*=0.085, TU: *P*=0.66, TL: *P*=0.43, WIK: *P*=0.0039), where female fish appeared to decrease their bottom distance over time whereas male fish showed little change across the trial. For distance from center (Fig. S2B), a main effect of interval was found for AB, TU, and WIKs (*P*'s=0.0004, TL: *P*=0.82), finding that these strains increased their distance from center over time. There were no interactions between sex and time interval (AB: *P*=0.92, TL: *P*=0.35, TU: *P*=0.92, WIK: *P*=0.92), but we found trends towards an effect of sex in TU and WIKs (AB: *P*=0.35, TL: *P*=0.35, TU: *P*=0.088, WIK: *P*=0.091) where female fish spent more time closer to the center of the tank than male fish, consistent with what was observed in the overall data (Fig. 2B). Finally, for distance travelled (Fig. S2C), there was an increase in locomotor activity over time in AB, TU, and WIK fish (AB: *P*=0.0004, TL: *P*=0.11, TU: *P*=0.0008, WIK: *P*=0.0004). Consistent with the overall data, there were also main effects of sex in all strains except TLs, where there was a trend (AB: *P*=0.00072, TL: *P*=0.098, TU: *P*=0.0004, WIK: *P*=0.014). Only the TU fish had an interaction between time interval and sex where TU female fish increased their distance travelled over time, but male fish did not (AB: *P*=0.91, TL: *P*=0.35, TU: *P*=0.0006, WIK: *P*=0.71).

### Correlations between behavioral parameters

To determine the extent to which individual behavioral parameters captured distinct elements of exploratory behavior, and if there was any influence of sex and genetics on these relationships, we computed correlations between individual behavioral measures (Fig. 2E-J; Fig. S3). We used Spearman's rank correlation coefficient (ρ) to identify monotonic relationships because of the presence of several non-normally disturbed parameters (Fig. S1). As expected, we found that distance travelled was consistently positively correlated with percent explored across all strains and sexes (Fig. 2J; Fig. S3F). We also found consistent positive correlations between bottom distance and percent explored (Fig. 2G; Fig. S3C), which is in line with the idea that a higher bottom distance is associated with an increased willingness to explore. However, bottom distance did not correlate consistently with distance travelled (Fig. 2F; Fig. S3B), suggesting that, despite positive correlations between distance travelled and percent explored, these two exploratory measures are capturing different aspects of exploration. Because thigmotaxis is usually described as a predator-avoidance behavior, we were surprised to find that bottom distance and center distance did not consistently correlate with each other (Fig. 2E; Fig. S3A): in three strain/sexes there was a clear negative correlation (female TLs and TUs, and male WIKs) where fish that swam nearer to the top also swam closer to the center, but in one group (male ABs) the opposite relationship was observed with no clear relationships in the remaining groups. Center distance was mostly negatively correlated with percent explored (Fig. 2I; Fig. S3E), but not universally so (TLs being the exception), largely consistent with the idea that fish that spend more time closer to the center of the tank also explore more of the tank.

### Identifying behavioral clusters

**Fig. 3.**
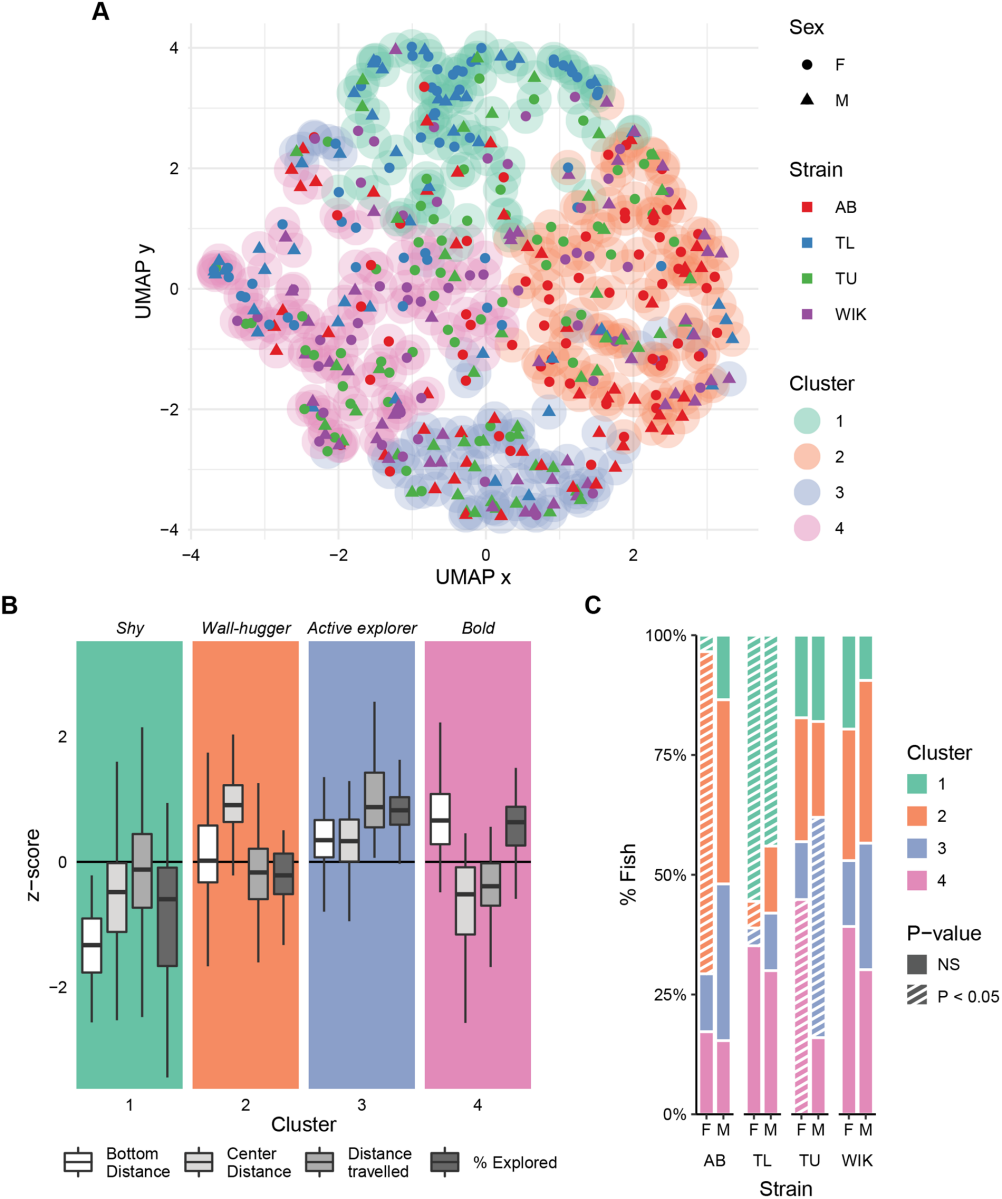
**Clustering of zebrafish exploratory behavior during initial exposure to the tank.** (A) Two-dimensional representation of the four-dimensional behavioral space using a uniform manifold approximation (McInnes et al., 2020preprint). Clusters (outer circles) are derived from Louvain community finding applied to a k-nearest neighbor network using 426 fish. Each point is an individual fish where the shape represents the sex (circle: female, triangle: male), inner color the strain, and outer color the behavioral cluster. (B) Individual behaviors associated with each cluster as box plots indicating median (center line), interquartile range (box ends), and hinge±1.5 times the interquartile range (whiskers). (C) Percentage of fish that fall into each cluster across strain and sex. Striped bars (*P*<0.05) represent over/under representation using randomized permutation tests and FDR corrections. AB, female: *n*=58, male: *n*=52; TL, female: *n*=54, male: *n*=50; TU, female: *n*=58, male: *n*=50; WIK, female: *n*=51, male: *n*=53.

Given the presence of non-normal behavioral distributions (Fig. S1) and variability in the relationship between different exploratory parameters (Fig. 2E-J), we hypothesized the presence of multiple behavioral clusters in our data set. To test this, we built a k-nearest neighbor network and applied the Louvain community detection algorithm to identify clusters (Blondel et al., 2008). Because none of the individual behavioral measures showed consistently high correlations across all strains and sexes (Fig. 2E-J), we used all four parameters (bottom distance, center distance, distance travelled, and percent explored) in calculating nearest neighbor distances. To determine ‘k’ for building the network, we explored a range of values and chose a value (k=114) that optimized internal clustering metrics and was robust to small deviations in k (Fig. S4). This resulted in the identification of four distinct behavioral clusters (Fig. 3A).

An examination of the behaviors associated with each of the clusters reveals a range of behavioral profiles that include traditional descriptions of bold and shy (Fig. 3B). Fish in the shyest cluster were lowest in both bottom distance and percent exploration (Fig. 3B; cluster 1) whereas fish in the bold cluster spent the most time near the top and center of the tank while also exploring more of the tank than average (Fig. 3B; cluster 4). Bold fish were also amongst the lowest in distance travelled, suggesting that they were not revisiting many parts of the tank. Two ‘mixed’ clusters were also identified: cluster 2 where fish were near average on all measures except for center distance where they spent most of their time near the periphery, a group we call ‘wall-huggers’. In cluster 3, fish were above average in distance travelled and percent explored, and also spent more time towards the periphery of the tank than bold fish; we call this group ‘active explorers’.

### Behavioral clusters across sex and strain

To determine whether fish from a given strain and sex were over- or underrepresented in behavioral clusters, we computed *P*-values using permutation resampling and FDR corrections for multiple comparisons (Fig. 3C). We found that, for the shy cluster (cluster 1), TL fish, irrespective of sex, were overrepresented (female: *P*=0.0012, male: *P*=0.002), whereas the wall-huggers (cluster 2) had overrepresentation of female AB fish (*P*=0.0013) and underrepresentation in female TLs (*P*=0.0013). Consistent with our finding that males, on average, swam more than females, more male fish were in the active explorers group (cluster 3), although overrepresentation was only significant in male TU fish, with a trend in ABs (males: AB: *P*=0.063, TL: *P*=0.32, TU: *P*=0.0013, WIK: *P*=0.35), and underrepresentation only significant in female TLs (females: AB: *P*=0.28, TL: *P*=0.0055, TU: *P*=0.28, WIK: *P*=0.49). Finally, in the boldest cluster (cluster 4), females outnumbered males in all strains but there was only significant overrepresentation in TU fish (female TU: *P*=0.038).

### Habituation over 2 days

All 426 fish used for generating clusters were exposed to the tank on 2 consecutive days, allowing us to determine whether their behavior remained consistent across repeated exposures. First, we analyzed individual exploratory behaviors with permutation paired *t*-tests and FDR corrections (Fig. S5). We found that AB and TL fish increased their bottom distance during the second exposure, with a trend towards an increase in female WIK fish (Fig. S5A; female AB: *P*=0.0085, male AB: *P*=0.012, female TL: *P*=0.0016, male TL: *P*=0.0040, female TU: *P*=0.62, male TU: *P*=0.58, female WIK: *P*=0.053, male WIK: *P*=0.62). Thigmotaxis (center distance) also increased in several groups: Female ABs and WIKs, with trends towards an increase in male ABs, but a decrease in TL males (Fig. S5B; female AB: *P*=0.014, male AB: *P*=0.063, female TL: *P*=0.38, male TL: *P*=0.076, female TU: *P*=0.12, male TU: *P*=0.48, female WIK: *P*=0.046, male WIK: *P*=0.96). Distance travelled did not change in any fish (Fig. S5C; female AB: *P*=0.30, male AB: *P*=0.46, female TL: *P*=0.30, male TL: *P*=0.11, female TU: *P*=0.13, male TU: *P*=0.64, female WIK: *P*=0.64, male WIK: *P*=0.34), and in percent explored there was a decrease in AB males and increase in TL females (Fig. S5D; female AB: *P*=0.16, male AB: *P*=0.037, female TL: *P*=0.037, male TL: *P*=0.38, female TU: *P*=0.83, male TU: *P*=0.32, female WIK: *P*=0.32, male WIK: *P*=0.28). Taken together, the changes in behavior during the second exposure were mixed, with some changes indicating an increase in behaviors associated with boldness, like bottom distance, and others an increase in putative shy behaviors, like thigmotaxis. However, given the lack of correlation between center and bottom distance (Fig. 2E), it is not clear that thigmotaxis should be interpreted as a shy behavior.

### Behavioral cluster consistency over 2 days

**Fig. 4.**
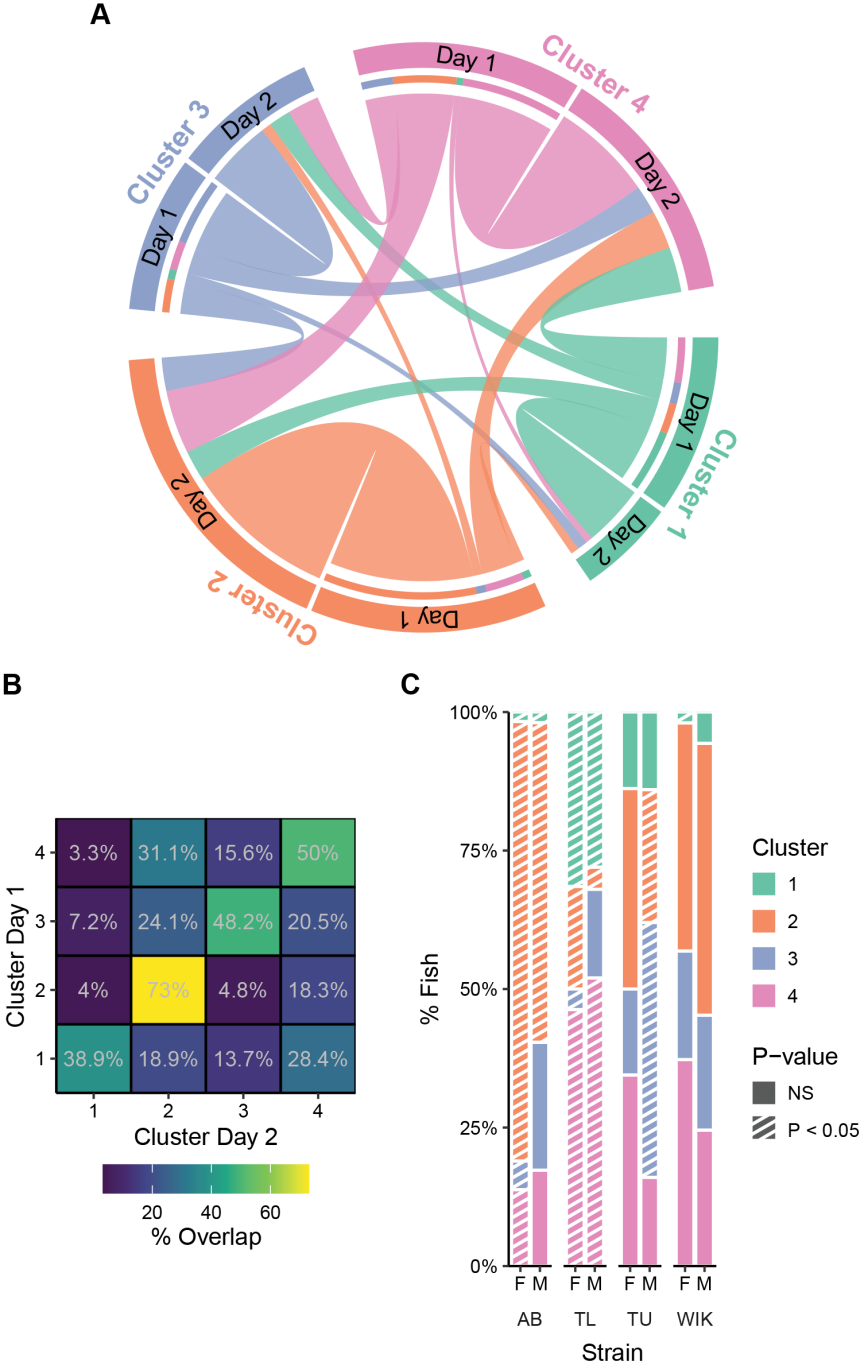
**Clusters across two consecutive exposures to the tank.** (A) Chord diagram indicating how cluster membership changes from day 1 to day 2 of novel tank exposure. (B) Percent overlap for each cluster from day 1 to day 2. (C) Percentage of fish that fall into each cluster across strain and sex on the second day of exposure to the novel tank. Striped bars (*P*<0.05) represent over/under representation using randomized permutation tests and FDR corrections. AB, female: *n*=58, male: *n*=52; TL, female: *n*=54, male: *n*=50; TU, female: *n*=58, male: *n*=50; WIK, female: *n*=51, male: *n*=53.

To determine if the behavioral clusters we identified remained consistent across days, we used exploratory data from the second day of exposure to the novel tank to assign fish to the clusters uncovered on the first day (Fig. 4A). We found that 54% of fish fell into the same behavioral cluster on days 1 and 2 (*P*=0.0001; permutation average=26.5% overlap). The extent of behavioral consistency depended on the cluster identity. Fish in cluster 2 (wall-huggers) on day one had the highest consistency (73%) whereas shy fish (cluster 1) had the lowest consistency (38.9%, Fig. 4A,B). Transitions to the shy group on day 2 were also the least likely to occur while transitions between the other three clusters were roughly similar (except cluster 2 to 3). This decrease in the number of shy fish during the second exposure likely reflects habituation to the tank (Fig. S5).

To determine if there were cluster differences across strain and sex on day 2 (Fig. 4C) we used FDR corrected permutation resampling. We found that, as on day 1, AB females were overrepresented in the wall-huggers group (cluster 2; *P*=0.0016) but were now also joined by their male counterparts (*P*=0.031). Both TL male and female fish were overrepresented in the shyest cluster (female TL: *P*=0.0016, male TL: *P*=0.010), but now a significant portion of male and female TL fish were also in the boldest cluster (female TL: *P*=0.029, male TL: *P*=0.0038). TU females were no longer overrepresented in the boldest group (*P*=0.69) and TU males maintained their overrepresentation in cluster 3 (active explorers, *P*=0.0016). Finally, as on day 1, neither female nor male WIKs were overrepresented in any cluster on day 2.

### Individual exploratory behaviors during multiple exposures to the tank (days and weeks)

Some exploratory behaviors have been found to habituate over several days of exposure to a tank (Wong et al., 2010). Thus, in a new cohort of AB and TU fish, exploratory behavior was measured over five consecutive days of exposure. Initially, we examined how individual exploratory behaviors changed using 5×2 (day×sex) mixed permutation ANOVAs to assess significance. For bottom distance in AB fish (Fig. S6A), there was no effect of day (*P*=0.19), sex (*P*=0.14) or an interaction (*P*=0.67), whereas TU fish increased their bottom distance over the 5 days (*P*=0.028), with no effect of sex (*P*=0.15) or an interaction (*P*=0.85). AB fish increased their center distance (Fig. S6B) over time (*P*=0.0001) with a trend towards an effect of sex where females appeared to spend more time towards the periphery than males (*P*=0.08) and no interaction (*P*=0.37). TU fish had a trend towards an effect of day (*P*=0.094), and no effect of sex (*P*=0.18) nor an interaction (*P*=0.97). As before, male AB fish swam further than female fish (*P*=0.011) with no effect of day (*P*=0.75). There was an interaction between day and sex (*P*=0.026) where AB males swam less over time, and females more (Fig. S6C). In TU fish, males also swam more than females (*P*=0.041), and there was an effect of day (*P*=0.0023) and no interaction (*P*=0.77) as both sexes increased their distance travelled over time. Finally, for percent explored (Fig. S6D), in AB fish there was no effect of sex (*P*=0.33) or day (*P*=0.87), but there was a trend towards an interaction (*P*=0.074) where female, but not male, fish appeared to slightly decrease their percent explored over time. In TU fish, animals increased their exploration over days (*P*=0.012), with a trend towards males exploring more than females (*P*=0.057), and no interaction of day and sex (*P*=0.17).

In a separate cohort of TU fish, we also examined individual exploratory behaviors over 10 weeks of biweekly (every other week) exposures to the tank (Fig. S7). We used 6×2 (week×sex) mixed permutation ANOVAs to assess significance. For bottom distance (Fig. S7A), we found no effect of week (*P*=0.96) or sex (*P*=0.25) but there was an interaction (*P*=0.0020) where female fish increased, and males decreased, their distance from bottom across weeks. For center distance (Fig. S7B), there was a trend towards an effect of week (*P*=0.059), and no effect of sex (*P*=0.22) or an interaction (*P*=0.69). For both distance travelled (Fig. S7C) and percent explored (Fig. S7D), there were main effects of sex (*P*=0.0003 and *P*=0.014, respectively) where, as we saw before, male fish swam further, and explored more of the tank, than female fish. There were also main effects of week (*P*=0.0001 and *P*=0.0004, respectively) and no interactions (*P*=0.78 and *P*=0.34, respectively), where both female and male fish increased their exploratory behaviors during repeated exposures.

### Behavioral cluster consistency over multiple exposures

**Fig. 5.**
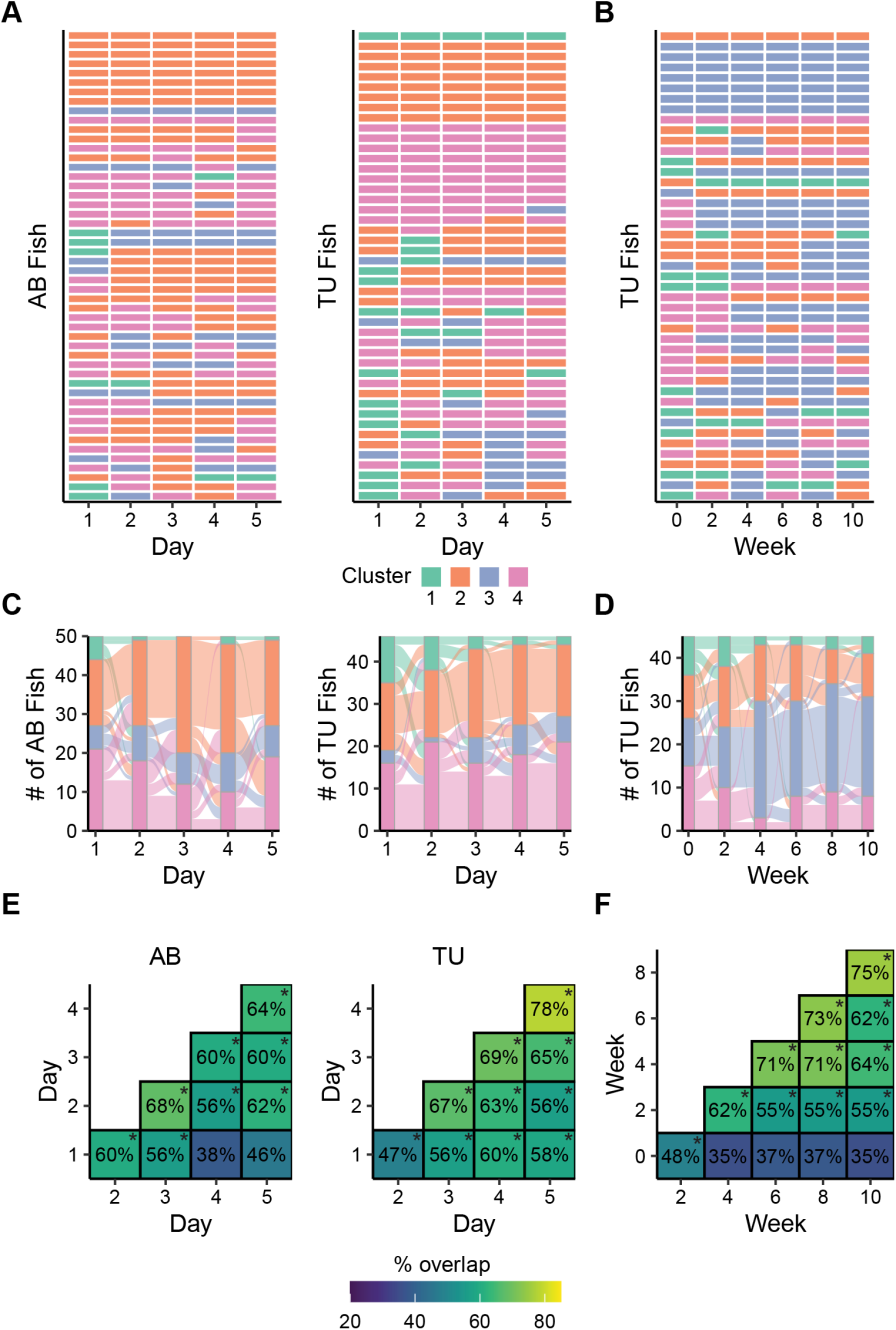
**Behavioral consistency of exploratory behavior across days and weeks.** (A) Cluster membership of AB (female: *n*=26, male: *n*=24) or TU (female: *n*=21, male: *n*=25) fish that were exposed to the tank on 5 consecutive days. (B) Cluster membership of TU (female: *n*=23, male: *n*=22) fish that were exposed to the tank every other week for 10 weeks. (C) Transitions between clusters across 5 consecutive days of exposure to the tank in AB and TU fish. (D) Transitions between clusters across six biweekly exposures to the tank in TU fish. (E) Percent of overlapping clusters in AB and TU fish across consecutive exposures to the tank across 5 days. (F) Percent overlapping clusters in TU fish across biweekly (every other week) exposures to the tank. *P<0.05 using randomized permutation test and FDR corrections.

Next, we asked whether the behavioral clusters of individual animals over days or weeks remained consistent (Fig. 5). Across the exposures we found that exploratory behavior of approximately 50% of animals fell into the same cluster on at least 4 out of 5 days (Fig. 5A) or 4 of 6 biweekly exposures (Fig. 5B). To determine if the consistency across time was greater than chance, each animal was assigned an overlap score: the sum of pair-wise overlaps across consecutive exposures to the tank. For the daily data this score ranged from 1 (only one pair of days overlapped) to 10 (all pair-wise overlaps), for the biweekly data it ranged from 2 to 15. During 5 days of exposure the average overlap scores for AB and TU fish were 5.7±2.5 and 6.2±3.2 (mean±standard deviation), respectively, both of which were significantly higher than the overlap scores from random resampling (permutation mean±standard deviation: 3.5±0.2 and 3.3±0.2, respectively; P's=0.0002). For the biweekly data, the average overlap score was 8.4±4.0, which was significantly higher than chance (4.6±0.2, *P*=0.0002). Interestingly, although most animals had scores higher than the permutation average, a small subset of animals were ‘consistently inconsistent’, with their exploratory behavior falling into all four clusters across days or weeks.

In both the daily and biweekly data, we found that the number of animals falling into the shyest cluster (cluster 1) decreased over time (Fig. 5C,D). In addition, in AB fish, the proportion of animals in clusters 2 (wall-huggers) and 4 (bold) vacillated across days with little change in the active explorer group (cluster 3). During daily exposures in TU fish, the relative proportions between the three non-shy clusters remained approximately equal throughout the experiment. During the biweekly exposures, most fish transitioned into the active explorer group by the end of fourth week and remained there throughout the experiment.

Finally, we examined how much cluster overlap there was between each of the 5 days, or weeks, of the experiment (Fig. 5E,F). During daily exposures, we found that cluster consistency was generally above chance, particularly after the second exposure, which likely reflects habituation to the tank. In daily exposures to the tank in both AB and TU fish, cluster overlap was mostly above 60% after the second day (Fig. 5E). In TU fish, this overlap increased to nearly 80% after 5 days of exposure. During the biweekly exposures (Fig. 5F), cluster overlap didn't increase markedly until the third exposure (week 4) where it then remained relatively high (62-75%).

## DISCUSSION

**Fig. 6.**
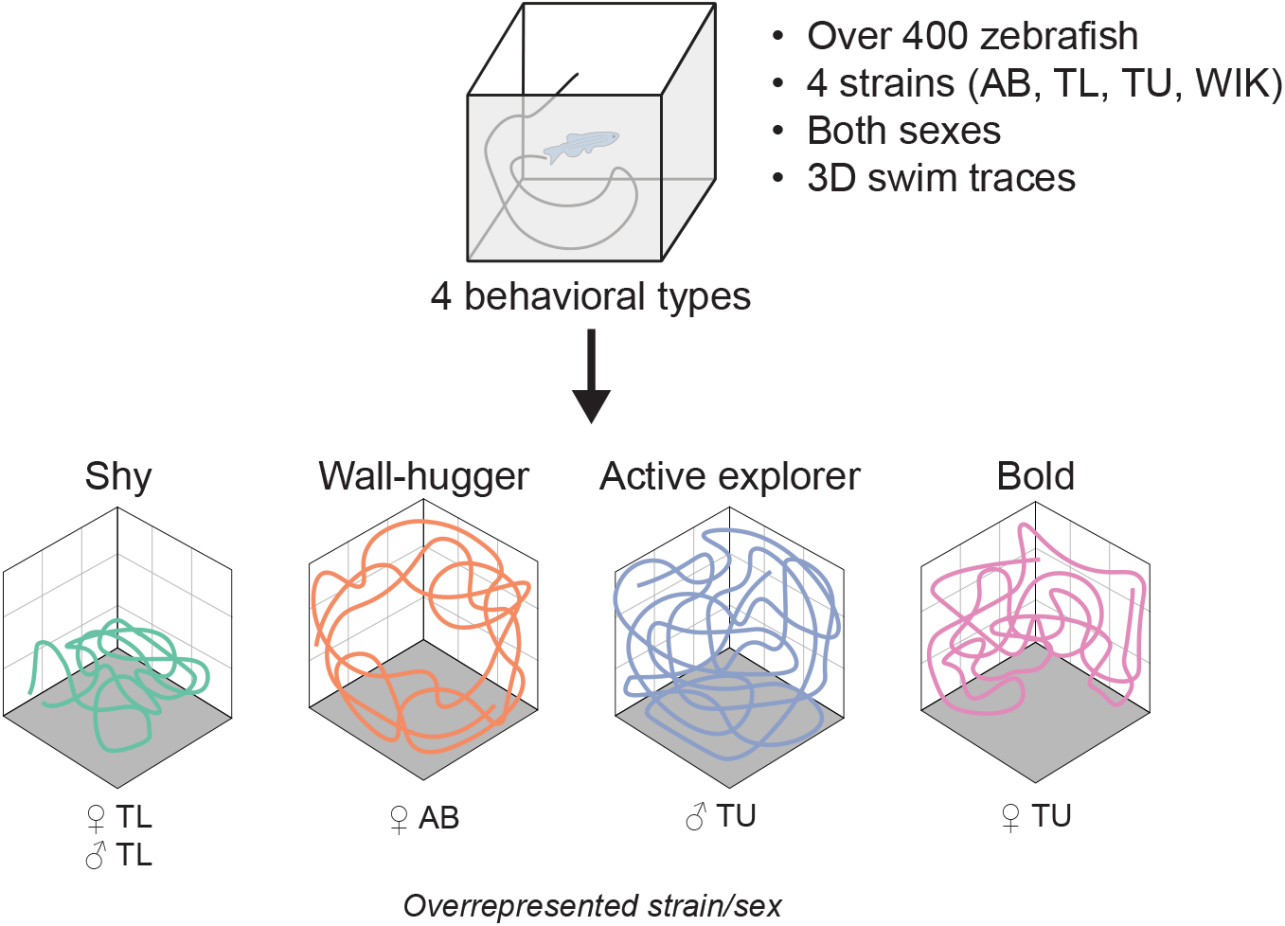
Summary of the findings in the paper along with the strains/sexes of fish that were overrepresented in the different clusters in response to the first exposure to the tank.

By applying an unbiased approach to the clustering of three-dimensional swim traces from over 400 zebrafish, we found that exploratory behavior stratifies into four distinct behavioral clusters. These profiles included previously described bold and shy behaviors as well as two novel behavioral types we call wall-huggers and active explorers (Fig. 6). Notably, these individual differences in fish behavior were consistent over days and weeks, one of the key hallmarks of personality. Although there were few strain–sex interactions on individual behaviors, the distribution of clusters varied considerably across strain and sex, suggesting biological modulation of these behavioral clusters.

Studies that have examined individual differences in exploratory behavior typically assume a bimodal distribution of bold versus shy (Oswald et al., 2012; Thörnqvist et al., 2019; Toms et al., 2010). Here, enabled by our large dataset of three-dimensional behavior, we tested this assumption. Using an unbiased unsupervised machine learning approach, instead of two categories, we found four behavioral subtypes (shy, wall-huggers, active explorers, and bold). Cluster 4 was the boldest cluster, where fish behavior corresponded to traditional descriptions of boldness: above average time in the top and center of the tank and greater exploration. The behavior of fish in cluster 1 was designated as shy because these fish spent most of their time near the bottom of the tank. Interestingly, in this shy group, behaviors like center distance and percent explored exhibited a high degree of variability, suggesting the potential presence of additional subgroups that would require an even larger data set to uncover.

Of the novel behavioral clusters we uncovered, we designated cluster 2 ‘wall-huggers’ because these fish spent most of their time towards the periphery of the tank and were average on all other parameters. We do not refer to this group as ‘shy’ because, although thigmotaxis has been interpreted as a predator avoidance or anxiety-like behavior in zebrafish (Kalueff et al., 2013), our findings do not support this interpretation. For example, we did not observe consistent negative correlations between center and bottom distance (Fig. 2E), as would be expected if these behaviors reflected the same underlying construct (i.e. predator avoidance). We also find that, unlike bottom dwelling, thigmotaxis increased over time within and between sessions of the novel tank test (Figs S2B and S6B), the opposite of the habituation we would expect to see if thigmotaxis was an anxiety-like or predator avoidance behavior. Our findings are similar to what has been observed in other studies that examined thigmotaxis over time and/or alongside other avoidance behaviors in adult fish (Blaser et al., 2010; Champagne et al., 2010; Rosa et al., 2018; Shams et al., 2015; Wong et al., 2012). It may be that the interpretation of thigmotaxis in zebrafish has been unduly influenced by findings in rodents where its importance for predator avoidance is clearer (Champagne et al., 2010; Simon et al., 1994). Thus, we propose that thigmotaxis in adult zebrafish should not be interpreted in the context of predator avoidance or anxiety until its relevance for fish can be clarified.

We called cluster 3 ‘active explorers’ because these animals had the highest distance travelled and percent explored. Given their elevated swim distance, this group of fish may correspond to what has been referred to as low stationary, or proactive, zebrafish in other work (Baker et al., 2018; Wong et al., 2012). Compared to the bold cluster, active explorers have similar levels of bottom distance and percent exploration, but a notable elevation in distance travelled and time near the periphery. Thus, during exploration, these animals may be less efficient in their exploration or exhibiting greater home base behavior as they revisit certain parts of the tank (Rosemberg et al., 2011; Stewart et al., 2010). One possible physiological contributor to this behavior may be a higher resting metabolic rate, which has been found to correlate with activity levels (Biro and Stamps, 2010; Yuan et al., 2018).

Strain and sex had a significant influence on behavioral cluster identity. With respect to sex, during the initial exposure to the tank, female fish more often fell into the boldest cluster, although only TU females were statistically overrepresented. Male fish were more likely to be in the active explorer group, with TU males overrepresented, and TL females underrepresented. This latter finding likely reflects the fact that, on average, male fish tended to swim further, and explore more of the tank, than female fish. This increase in locomotor activity in male fish has been reported elsewhere (Ariyomo and Watt, 2015; Clayman et al., 2017; Philpott et al., 2012), although not universally so (Ampatzis and Dermon, 2016; Fontana et al., 2019; Rambo et al., 2017; Tran and Gerlai, 2013). We also found effects of strain, such as overrepresentation of TL fish in the shyest cluster. Sex by strain interactions were also evident, such as overrepresentation of AB females in the wall-huggers group. Many of these differences persisted into the second day of exposure with the most notable difference being that both AB male and female fish were now overrepresented as wall-huggers, and TL fish were overrepresented in both the bold and shy clusters.

The behavioral clusters we identified demonstrated consistency across days and weeks, supporting the idea that they are akin to personality types (Gosling, 2001). In our initial experiment, where fish were tested in the tank on 2 consecutive days, overlap was 54%, twice as high as would be expected by chance (∼25%). Upon repeated exposures to the tank over 5 consecutive days we found that, in TU fish, cluster overlap increased to as high as 78%, and that in both AB and TU fish over half of the fish were in the same cluster on 4 of the 5 days. We found similar effects during biweekly exposures in TU fish over 10 weeks. Overlap across days in AB fish was initially higher than TUs but did not increase as much with a substantial minority of fish vacillating between clusters 2 and 4 (wall-huggers and bold). This increase in overlap over days in TU fish is likely due, in part, to habituation (Wong et al., 2010) given that we found that there were main effects of day on several individual measures (Figs S6 and S7). The percent overlap we observed is not dissimilar to what has been observed in human personality research where weekly test/ret-test reliability across measures has a median of 0.83 and range of 0.71-0.91 (McCrae et al., 2011).

The present work also yielded a comprehensive account of how genetic background influences individual exploratory behaviors of zebrafish, adding to a rich, if inconsistent, literature. The primary strain difference on individual behaviors we observed was that TL fish differed from most other strains, demonstrating higher bottom dwelling, less thigmotaxis, and exploring less of the tank. Although strain differences in zebrafish behavior during the novel tank test have been reported (Egan et al., 2009; Maximino et al., 2013; Mustafa et al., 2019), only a few studies have compared widely used inbred strains. Vignet and colleagues (2013) found that AB fish exhibited more bottom dwelling than TU fish, and Audira et al. (2020) found no differences between AB, TL, and WIK fish in bottom dwelling or distance travelled, but that WIK fish exhibited greater thigmotaxis. Others have found AB fish to exhibit greater bottom dwelling than WIKs (Sackerman et al., 2010), or a decrease in bottom dwelling in WIK fish over 60 min, but not TU fish, with no difference in locomotor activity (Pannia et al., 2014). Differences between these studies, and the present work, may be attributable to unreported sex ratios, housing conditions, nuances of the behavioral task, or genetic drift such that inbred strains from different labs or suppliers may differ subtly, as has been observed in rodents (Taft et al., 2006). We attempted to address some of these challenges in our study. For example, housing conditions are known to influence zebrafish behavior in the novel tank test (Parker et al., 2012; Reolon et al., 2018), so we ensured consistent housing across our study: fish were placed in mixed sex pairs for one week prior to behavioral testing. This allowed us to maintain zebrafish identify over time while avoiding any effects of stress due to social isolation or tagging. To minimize genetic drift and maximize the likelihood our findings will translate to other labs, all fish were within two generations of breeders obtained from the Zebrafish International Resource Center where they maintain a much larger, and genetically diverse, population of animals. Nonetheless, given that inbred zebrafish lines are not isogenic (Nasiadka and Clark, 2012), there is no obvious way to ensure genetic similarity of lines across labs.

Some potential limitations of the present work are the number of behavioral parameters used to identify clusters and the size of our tank. Although we could have generated many more parameters, we limited our analysis to those most clearly related to exploratory behavior (i.e. position and activity). This decision may have limited our ability to detect more clusters. However, even with four parameters that could take on one of three states (low, medium, or high), there are already 81 possibilities. The tank we chose for testing was cube shaped (15 cm per side) filled with 2.5 L water to a depth of ∼12 cm. Although this allowed us to assess both bottom dwelling and thigmotaxis, it is smaller than what zebrafish would experience in the wild. Thus, it may be the case that our tank could have obscured the presence of different behavioral subtypes that might be more evident in a larger arena (Stewart et al., 2012).

Taken together, we found that the exploratory behavior of zebrafish goes beyond bold versus shy, stratifying instead into four different behavioral subtypes. This finding was enabled by recent advances in animal tracking and three-dimensional video capture that allowed us to scale up our behavioral assessment using inexpensive open-source tools. As would be expected of behaviors capturing personality, the clusters we identified were consistent over days and weeks and influenced by strain and sex. Future work will be needed to determine if these clusters are predictive of behaviors in other contexts and to identify the molecular and neural basis for these behavioral differences. Nonetheless, our findings suggest that animal behavior is more complex than is typically assumed and should be considered when examining individual differences in animal behavior.

## MATERIALS AND METHODS

### Subjects

Subjects were female and male AB, TU, WIK, or TL zebrafish 16-32 weeks of age. All fish used in experiments were bred and raised at Wayne State University and within two generations of animals obtained from the Zebrafish International Resource Center at the University of Oregon. Animals were kept on high density racks under standard conditions (temperature 26.5±0.5°C; water conductivity 500±10 μS, and a pH of 7.5±0.2) with a 14:10 light:dark cycle (lights on at 8:00AM). Fish were fed twice a day with a dry feed in the morning and brine shrimp (*Artemia salina*; Brine Shrimp Direct, Ogden, UT, USA) in the afternoon. Behavioral testing took place between 11:00 and 14:00.

Sex of fish was determined using three secondary sex characteristics: shape (prominent belly for females), color (males more pink/red in coloration), and presence of pectoral fin tubercles (present in males; McMillan et al., 2015). Following behavioral procedures, sex was confirmed by determining the presence or absence of eggs via dissection. Those animals that were assigned the wrong sex were removed from analysis (<3%). All procedures were approved by the Wayne State University Institutional Animal Care and Use Committee.

### Behavioral apparatus

Five-sided tanks (15×15×15 cm) were made from frosted acrylic (ShopPopDisplays, Woodland Park, NJ, USA) and open from above. Tanks were placed in an enclosure made of white plasticore to diffuse light and prevent the influence of external stimuli. D435 Intel RealSense™ cameras (Intel, Santa Clara, CA, USA) were mounted 20 cm above tanks to capture three-dimensional videos (Kuroda, 2018). D435 cameras capture three-dimensional videos using the synchronous capture of two video streams: a color stream (red/green/blue) and a depth stream. The depth stream is generated via stereoscopic imaging using the disparity between two infrared cameras. Firmware on the camera synchronizes capture of the two streams. Cameras were connected to a Linux workstation via high-speed USB cables (NTC distributing, Santa Clara, CA, USA) and video capture was controlled via Python scripts that are available upon request. Animals with videos that were not fully recorded due to malfunction were excluded from analysis.

### Behavioral procedures

One week prior to behavioral testing, fish were placed as male/female pairs into 2 L tanks. The tanks were divided in half with a transparent divider with two fish in each section and a total of four fish in each tank. This allowed us to maintain the identity of fish over days without isolation while also creating a consistent social environment across all animals. On days when behavior was assessed, fish were taken off housing racks and moved to the procedural space at least 1 h prior to behavioral testing. Following testing, fish sat for one hour before being returned to the housing racks. Experimental tanks were filled with 2.5 L of fish facility water and individual fish were placed in the tanks for 6 min while video was recorded for offline analysis. Tanks were rinsed between animals and water was replaced.

### Animal tracking

Fish were tracked in the color videos using DeepLabCut (Mathis et al., 2018). We tracked five points (head, trunk, and three points on the tail; Fig. 1C). Using ResNet 101, we initially trained the network on 160 frames equally divided across fish of all four strains and both sexes. We refined and improved our initial training by correcting outliers and including an additional 160 frames. After training, the test error for points identified with at least 10% likelihood was less than 3 pixels.

To obtain the z-coordinate of fish at each frame, we overlaid the tracked points at each color frame with the depth stream from the cameras. The depth stream underwent the following post-processing steps to increase accuracy (default parameters were used unless otherwise noted): a 3-pixel decimation filter was followed by a spatial filter with 2-pixel hole-filling and then a temporal filter with a persistency index of 4 (2 of 8 frames) was applied. The z-coordinate for the fish was identified for each frame based on a 4-pixel search radius around the tracked points starting with the trunk. If no point was identified from the depth stream, it was interpolated (typically<1% of frames). Z-coordinates were corrected for the diffraction of water by measuring 100 points of varying distance from the camera in the presence and absence of water and using the equation of a least-squares fit line.

### Exploratory behavioral parameters

To measure bottom distance, we calculated the equation for a plane along the bottom of the tank using least squares fit of 400 points. We then calculated the shortest distance between a point (the fish) and the plane. To measure center distance, we calculated how far fish were from a line made from points at the center top and center bottom of the tank. Percent of the tank explored was calculated by dividing the tank into 1000 evenly spaced voxels and calculating the number of unique voxels visited. Before calculating bottom distance, center distance, and distance travelled, traces were smoothed using a Savitzky–Golay filter with a length of seven frames and an order of three (Press and Teukolsky, 1990). For distance travelled, the Euclidean distance was calculated between points of successive frames.

### Behavioral clusters

We identified behavioral clusters using a Louvain community detection algorithm (Blondel et al., 2008) applied to a k-nearest neighbor network on our initial dataset of 426 fish during their first day of exposure to the tank. This was done by first standardizing individual behavioral parameters (bottom distance, center distance, distance travelled, and percent explored), and calculating a similarity score between each fish:

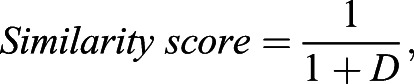
where D is the Euclidean distance between each fish in four-dimensional behavioral space. To determine the best k for building the network, we initially used a range of k's, applied the Louvain community finding algorithm to weighted, non-directed networks, and calculated internal clustering metrics (Calinski–Harabsz index, Caliński and Harabasz, 1974; Silhouette index, Rousseeuw, 1987; and Davies–Bouldin index, Davies and Bouldin, 1979). We chose k=114, which was in the middle of a regime that optimized internal clustering and was robust to small changes in k (Fig. S4).

To identify clusters in new data, for example the second day of novel tank exposure (Fig. 4) or fish exposed on consecutive days or biweekly (Fig. 5), we first standardized the new data using the parameters from the 426 fish exposed to the novel tank described above. Then, for each new data point, we assigned clusters based on the proportion of connections to its 41 nearest neighbors in the initial network where 41 is half the size of the smallest cluster identified.

### Coding and statistical analysis

Statistical analysis was performed using R version 4.1.2 (R Core Team, 2016) and visualized using ggplot2 (Wickham, 2015) and RColorBrewer (Neuwirth, 2014). Normality was assessed using the Shapiro–Wilks test. Because a considerable portion of the data was not normally distributed, we used permutation ANOVAs using the permuco package (Frossard and Renaud, 2021), and the RVAideMemoire (Hervé, 2021) for permutation *t*-tests. Multiple comparisons were corrected using a false discover rate (FDR) correction (Benjamini and Hochberg, 1995). We used packages cccd (Marchette, 2015) and igraph (Csardi and Nepusz, 2006) to build and analyze the k-nearest neighbor network, and ClusterCrit (Desgraupes, 2018) to assess internal clustering metrics. The chord diagram was made using circlize (Gu et al., 2014).

For all permutation tests, we resampled data 10,000 times without replacement and calculated the *P*-value as the proportion of experimental observations that were more extreme than the permutation observations. When multiple tests were performed, *P*-values were corrected using the FDR as indicated. To determine whether behavioral clusters were affected by strain and sex (Figs 3C and 4C), we resampled cluster assignment and calculated how many animals from each strain and sex fell into each cluster. For comparison of behavioral clusters across 2 days (Fig. 4C) we resampled cluster assignments from day 2. To determine if behavior was consistent across daily (5 day) or biweekly (every other week over 10 weeks) (Fig. 5A,B), we first calculated an overlap score for each fish that was the sum of the number of days or weeks that had overlapping clusters (ranging from 1 to 10 for 5-day data, 2 to 15 for biweekly data). We then took the average of these overlap scores and compared them to scores obtained from permutation resampling (without replacement) of cluster assignments. Five-day and biweekly percent overlap calculations and permutation tests were as described for the 2-day data except that resampling of cluster assignments was done individually for all days/weeks except the first day/week.

## Supplementary Material

Supplementary information
