# Supplementary material for: Beyond bold versus shy: Zebrafish exploratory behavior falls into several behavioral clusters and is influenced by strain and sex
Source: Biol Open. 2022 Aug 30;11(8):bio059443. doi: 10.1242/bio.059443 (PMC9450886; doi:10.1242/bio.059443)
Supplement: Supplementary information [file biolopen-11-059443-s1.pdf]

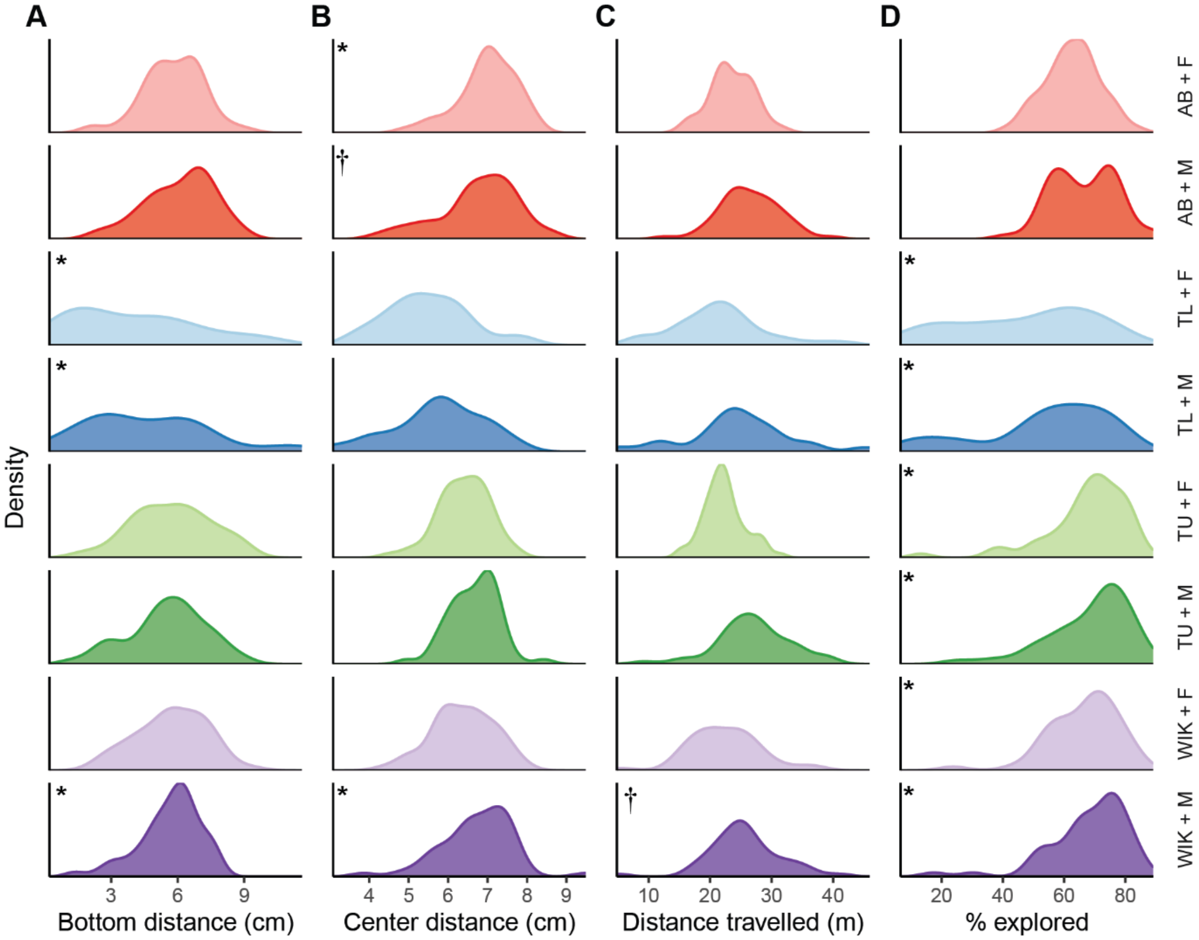

Fig. S1. Distribution of values for individual behavioral parameters across strain and sex. Distributions for **A**) bottom distance, **B**) center distance, **C**) distance travelled, and **D**) percent tank explored. * - $P < 0.05$, † - $P < 0.10$ Shapiro-Wilks test for normality, n = 50-58.

Biology Open • Supplementary information

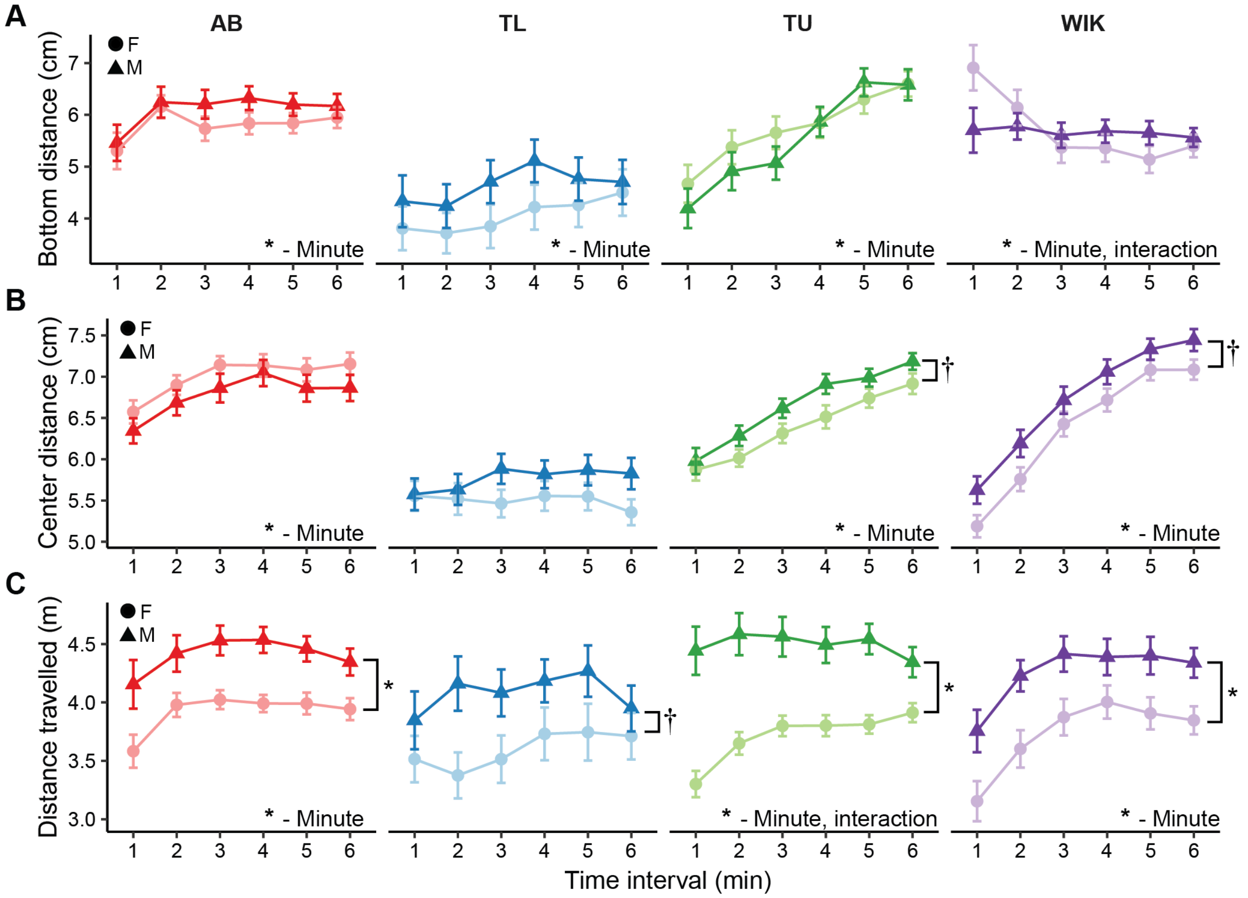

Fig. S2. Individual exploratory behaviors across six minutes during a single exposure to the tank. The effect of strain (color) and sex (shape, circle: female, triangle: male) across time for **A**) bottom distance, **B**) center distance, and **C**) distance travelled. * - P < 0.05, † - P < 0.10 for time interval (minute) or sex as indicated, n = 50-58. Data presented as mean ± SEM.

Biology Open • Supplementary information

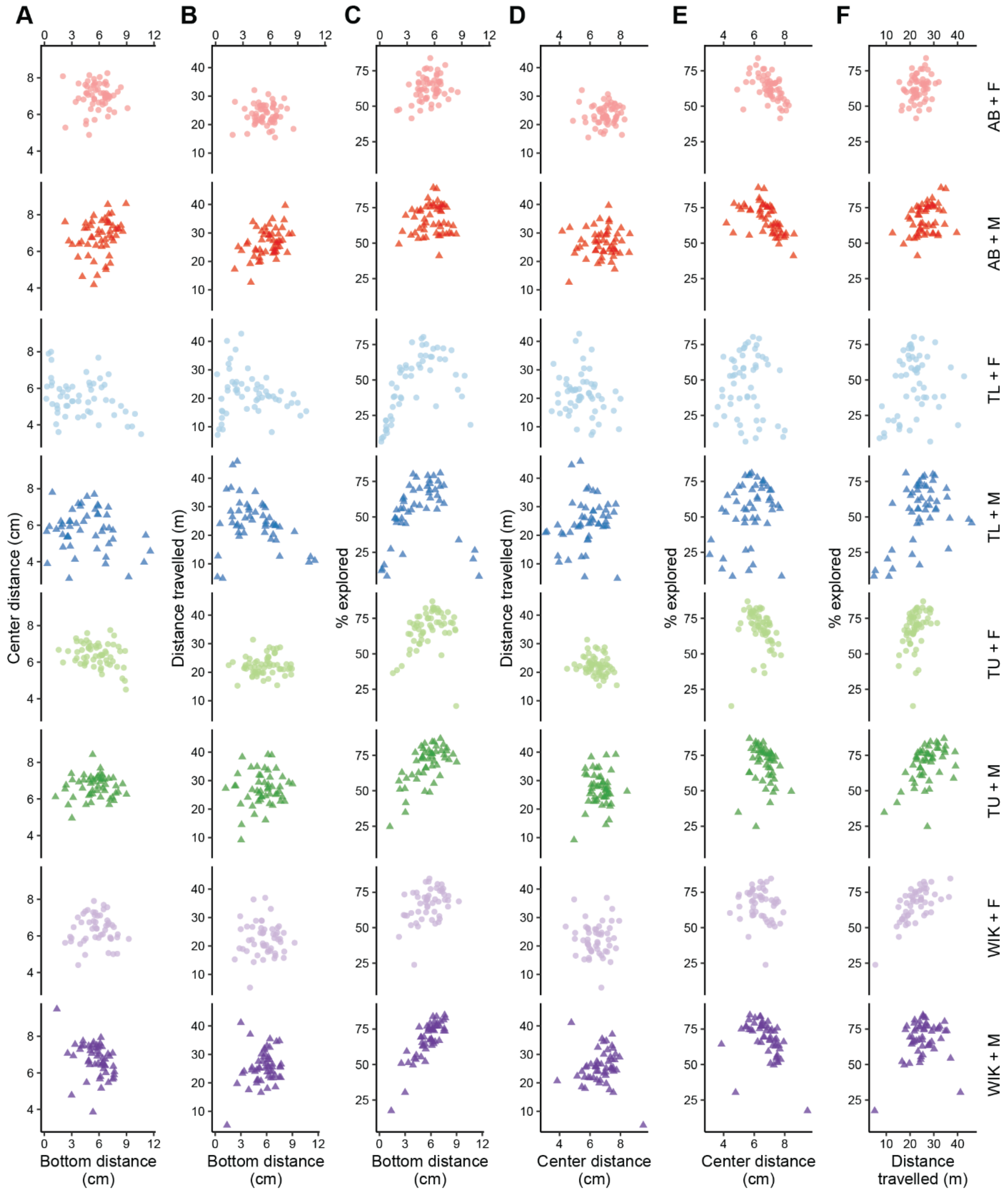

Fig. S3. Relationship between pair-wise individual behavioral parameters. Scatterplots across strain and sex of **A**) bottom distance versus center distance, **B**) bottom distance versus distance travelled, **C**) bottom distance versus percent explored, **D**) center distance versus distance travelled, **E)** center distance versus percent explored, and **F)** distance travelled versus percent explored. n = 50-58.

Biology Open • Supplementary information

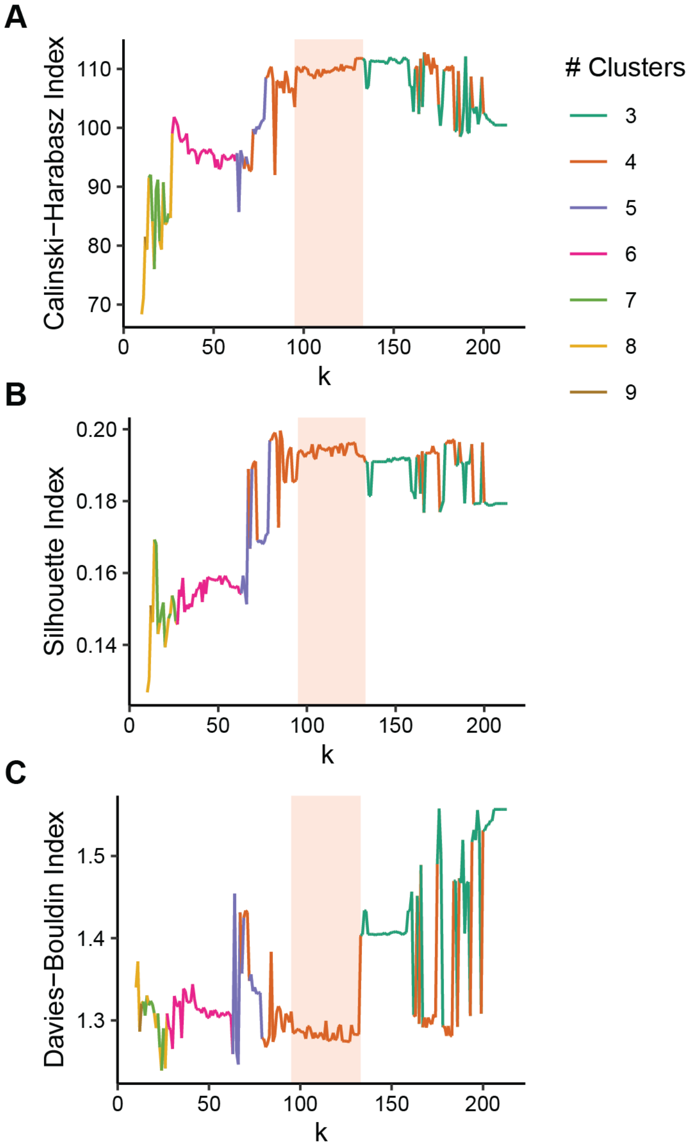

Fig. S4. Internal clustering metrics for knn Louvain clustering. The parameter k was varied for generating knn's and Louvain clustering followed by measuring the **A**) Calinski-Harbasz, **B**) silhouette, and **C**) Davies-Bouldin indices at each k. Area highlighted in red indicates region where clustering metrics were near optimal and robust to small changes in k.

Biology Open • Supplementary information

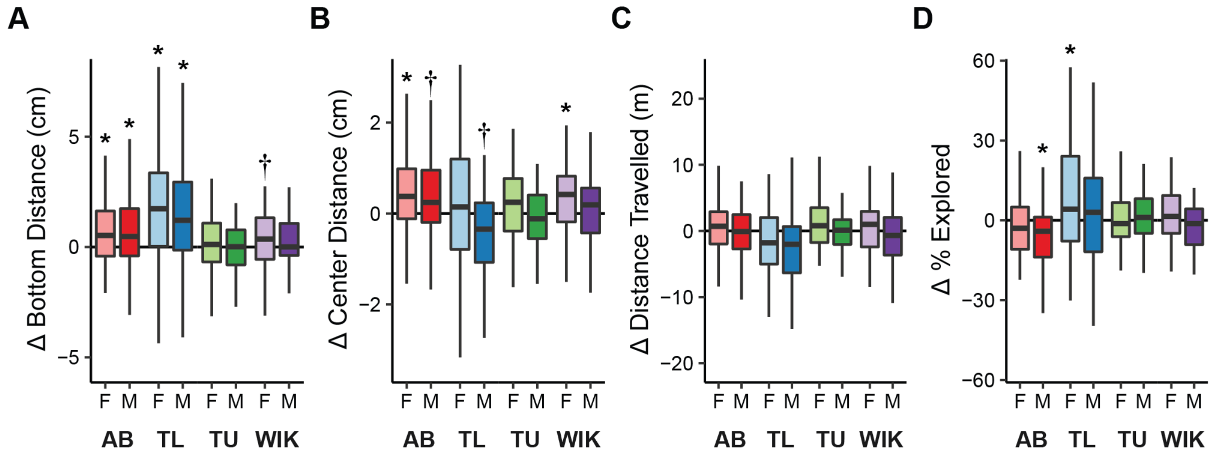

Fig. S5. Changes in individual exploratory behaviors during two consecutive exposures to the tank. The effect of strain and sex on changes (day 2 minus day 1) over two days in **A**) bottom distance, **B**) center distance, **C**) distance travelled, and **D**) percent explored. Boxplots indicate median (center line), interquartile range (box ends), and hinge ± 1.5 times the interquartile range (whiskers). * - $P < 0.05$, † - $P < 0.10$ compared to day 1, n = 50-58.

Biology Open • Supplementary information

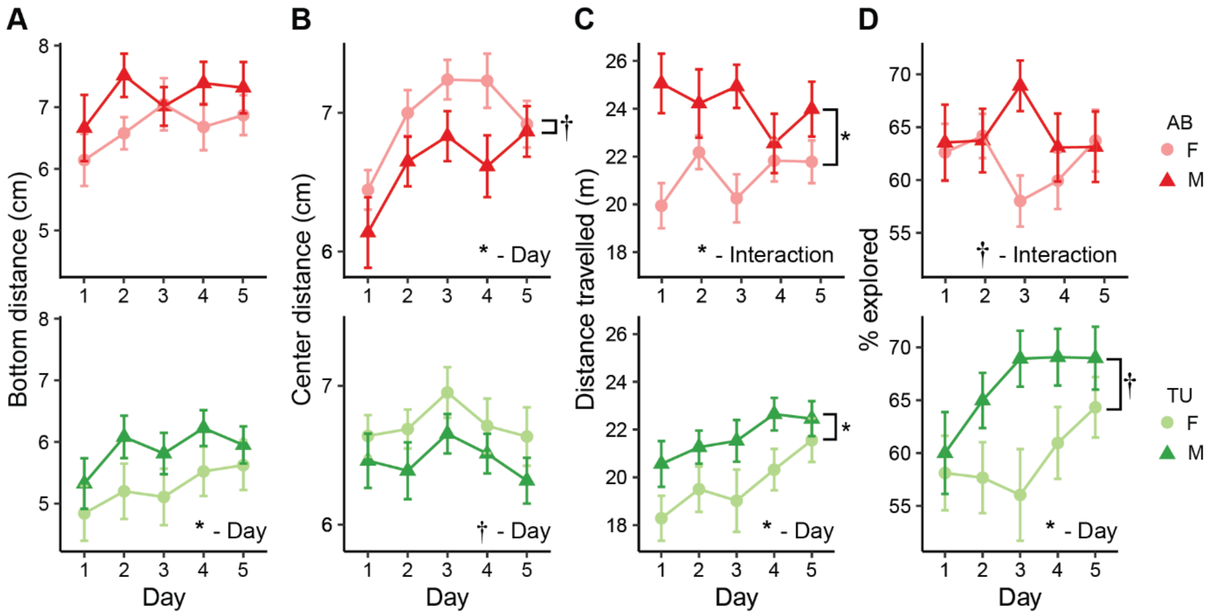

Fig. S6. Individual exploratory behaviors during five consecutive days of exposure to the tank. AB (top) and TU (bottom) fish were exposed to the tank on five consecutive days and **A**) bottom distance, **B**) center distance, **C**) distance travelled, and **D**) percent explored were measured. * - P < 0.05, † - P < 0.10 for day or sex and indicated, n = 21-26. Data presented as mean ± SEM.

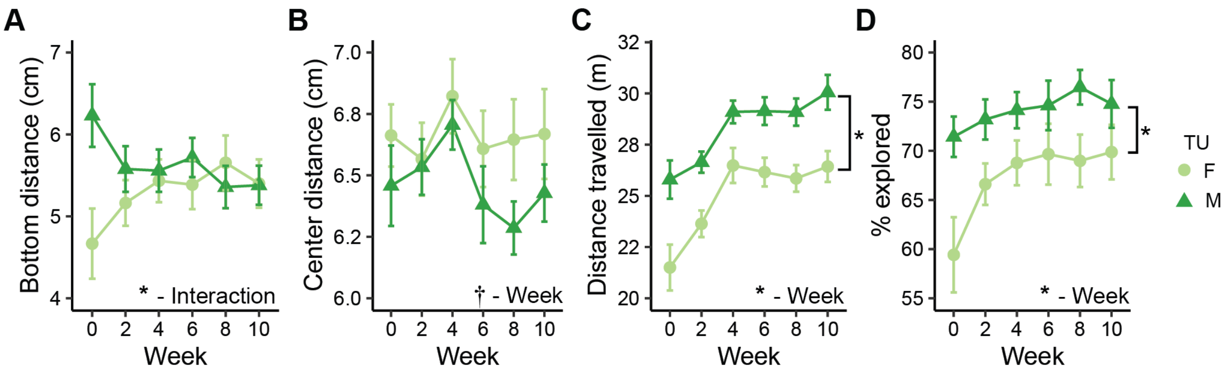

Fig. S7. Individual exploratory behaviors over ten weeks. TU fish were exposed to the tank every other week and we measured **A**) bottom distance, **B**) center distance, **C**) distance travelled, and **D**) percent explored. * - $P < 0.05$, † - $P < 0.10$ for week or sex as indicated, n = 22-23. Data presented as mean ± SEM.

Biology Open • Supplementary information
